# Mediator role of presence of meaning and self-esteem in the relationship of social support and death anxiety

**DOI:** 10.3389/fpsyg.2022.1018097

**Published:** 2022-12-05

**Authors:** Yuxin Huang, Ziyao Guan, Fang Yan, James A. Wiley, Nancy R. Reynolds, Siyuan Tang, Mei Sun

**Affiliations:** ^1^Teaching and Research Section of Clinical Nursing, Xiangya Hospital, Central South University, Changsha, Hunan, China; ^2^Xiangya School of Nursing, Central South University, Changsha, Hunan, China; ^3^School of Social Sciences, Faculty of Arts, Design and Architecture, University of New South Wales, Sydney, NSW, Australia; ^4^Family and Community Medicine and Institute of Health Policy Studies, University of California, San Francisco, San Francisco, CA, United States; ^5^School of Nursing, Johns Hopkins University, Baltimore, MD, United States

**Keywords:** COVID-19, death anxiety, presence of meaning, terror management theory, self-esteem, social support

## Abstract

**Introduction:**

Death anxiety has increased following the COVID-19 pandemic. Although terror management theory has suggested social support, presence of meaning and self-esteem functioned as death anxiety buffers, few existing works have explored the mechanism of how social support, presence of meaning, and self-esteem buffer death anxiety. To identify these mechanisms is the aim of this study.

**Methods:**

Our cross-sectional study was conducted with 1167 people in China from 19 May 2020 to 1 June 2020 during the COVID-19 outbreak. The average age of participants was 26 years. Data were by questionnaire, including demographic information, the Templer's Death anxiety scale, the multidimensional scale of perceived social support, the presence of meaning scale, and the Rosenberg self-esteem scale.

**Results:**

Results using structural equation modeling showed presence of meaning and self-esteem fully mediated the relationship between social support and death anxiety, respectively and sequentially. The proposed model showed good fit of indices: χ^2^ = 243.384, df = 58, *p* < 0.001; CFI = 0.968, TLI = 0.954, RMSEA = 0.052, SRMR = 0.044.

**Discussion:**

This study demonstrates significant mediator roles of presence of meaning and self-esteem in the relationship of social support and death anxiety. Multi-component interventions are needed to manage death anxiety by targeting increasing social support, presence of meaning and self-esteem and increasing presence of meaning and self-esteem when social support is diminished in the pandemic.

## Introduction

The COVID-19 (Coronavirus Disease 2019) has caused tremendous damage throughout the world (Galea et al., 2020). According to the World Health Organization (WHO), 239,437,517 confirmed cases of COVID-19 including 4,879,235 deaths globally were reported, among which 125,162 confirmed cases and 5,695 deaths happened in China by 15 October 2021 (World Health Organization, 2021a,b). With the widespread disease and deaths caused by the COVID-19 and news about deaths and new cases reported, death anxiety has been accentuated and has become a public issue (Chen et al., 2020; Kavakli et al., 2020).

Terror Management theory (TMT) states that death anxiety is aroused from death-related thoughts and exists in all human beings because human intellectual ability allows them to realize the vulnerability and mortality of their lives and as a consequence produces existential concerns (Greenberg et al., 2014). A high level of death anxiety was observed in the general population as the COVID-19 pandemic continued (Chen et al., 2020; Kavakli et al., 2020). Death anxiety is thought to be associated with mental disorders, such as panic, generalized anxiety, and depressive disorders (Iverach et al., 2014; Lee et al., 2020). Given the nature of Chinese traditional culture, people are maladapted to death-related thoughts because they see death as something unfortunate (Yin et al., 2017). Mortality was made salient by the pandemic. Some observers argue that the death anxiety level in China may be higher than that of any other country (Yin et al., 2017; Khajoei et al., 2022). Considering the mental disorders that death anxiety could cause, looking for protectors against death anxiety in the context of COVID-19 pandemic is an important reflection at improving mental health of the general population.

According to the TMT (Rosenblatt et al., 1989), a leading theory explains the influence of death on individual behavior in the field of social psychology, social support, presence of meaning, and self-esteem which serve as death anxiety buffers when mortality is made salient (Rosenblatt et al., 1989; Schmeichel et al., 2009; Plusnin, 2018; Perach and Wisman, 2019). Social support is the support received from social network, namely the support that comes from family members, friends, and significant others (Zhou et al., 2015). Presence of meaning is a cognitive dimension that emphasizes the outcome of having meaning in life (Steger and Frazier, 2005; Li et al., 2021a,b). Self-esteem is the extent to which one holds positive views of oneself (Schmeichel et al., 2009). Previous research based on TMT focused on how each of these factors buffers the influence of death anxiety on individual behavior or well-being (Plusnin, 2018; Cox et al., 2021). Less research has focused on the interrelationships between social support, presence of meaning, self-esteem, and death anxiety. Consequentially, only one-sided interventions with limited effect have been developed to manage death anxiety and its effect on individual behavior or well-being (Greenberg et al., 2014; Breitbart et al., 2018). To develop targeted multicomponent interventions, the interrelationships among social support, presence of meaning, self-esteem, and death anxiety should be understood (Greenberg et al., 2014; Plusnin, 2018).

The TMT suggests (Schmeichel et al., 2009; Martela and Steger, 2016; Barnett et al., 2019; Dewitte et al., 2019; Harris and Orth, 2020) that social support, presence of meaning, and self-esteem are protective factors against death anxiety (Zhang et al., 2019; Bibi and Khalid, 2020). Apart from the TMT, other theoretical accounts predict the associations between social support, presence of meaning, and self-esteem. Attachment theory mentions that satisfying relationships with the attachment figures provide a sense of order and coherence, which are the main components of the presence of meaning in life (Martela and Steger, 2016; Dewitte et al., 2019; Li et al., 2021b). Additionally, positive psychology has pointed out the important role of the presence of meaning for mental health (Li et al., 2021b), and self-esteem is an important indicator of mental health (Schmeichel et al., 2009).

Previous researches on the interrelationships among social support, self-esteem, and presence of meaning show that these factors are positively correlated with each other (Zhang et al., 2019; Poudel et al., 2020; Li et al., 2021b). Moreover, self-esteem and presence of meaning were positively affected by social support (Poudel et al., 2020; Li et al., 2021b), suggesting that individuals with a higher social support tend to view themselves positively and live a more meaningful life (Li et al., 2021a). A previous study reported that presence of meaning is strongly associated with subjective well-being (Li et al., 2021a) and is an important source of self-esteem (Zhang et al., 2019). Moreover, presence of meaning can be considered as an external cause because it develops from a set of validated and credible norms and beliefs, while self-esteem can be seen as an internalized buffer (Lu et al., 2019; Zhang et al., 2019). It is possible that the achievement of meaning from a strong connection to cultural context may also support the internal development of self-esteem (Martela and Steger, 2016). Presence of meaning and self-esteem may mediate the relation between social support and death anxiety, individually and through a longer chain of connections (Lu et al., 2019).

Insufficient social interactions from social and physical distancing due to self-quarantine policies lead to a diminished social support (Labrague et al., 2021). The absence of an important buffer against death anxiety makes the examination of the relationships among social support, presence of meaning, self-esteem, and death anxiety an important research priority.

**Figure 1:**
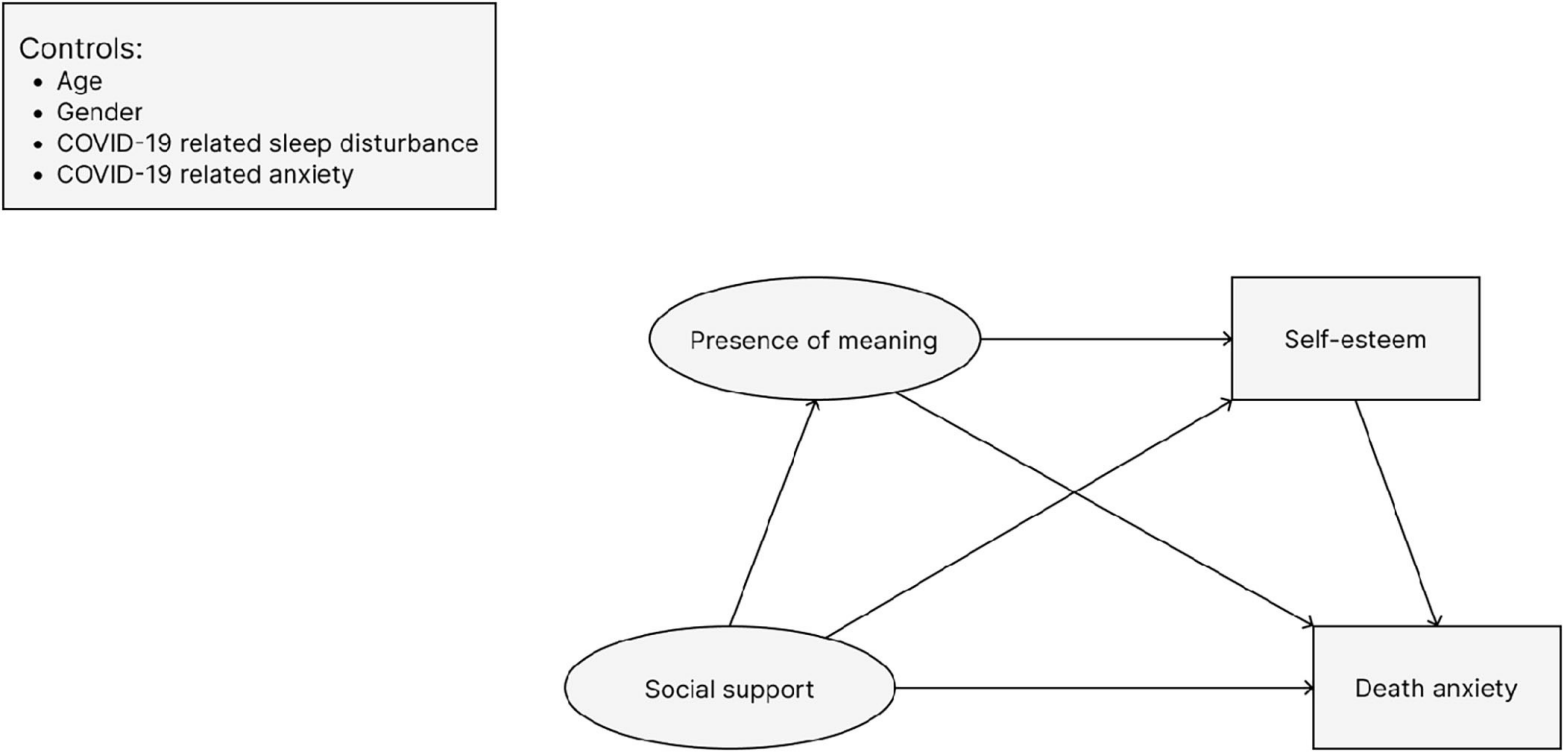
Hypothesized structural equation modeling. Circles indicate latent variables, while rectangles represent observed variables.

This study aimed to examine the relationships of social support, presence of meaning, self-esteem, and death anxiety. It was hypothesized that (H1) social support is protective against death anxiety and thus as social support increases, we should observe a corresponding decrease in death anxiety. (H2) We expect social support to encourage both the presence of meaning and self-esteem as buffers against death anxiety. (H3) As mediating factors, meaning is antecedent to self-esteem, so that a chain of influence on death anxiety begins with social support and flows to presence of meaning and then to self-esteem. Additionally, a more nuanced look at which specific path is the most effective way from social support to buffer death anxiety is important to determine which component should be the main focus when developing multicomponent interventions to reduce death anxiety. We formulated the structural equation model (SEM) displayed in Figure 1 based on the TMT and the literature reviewed above. The circles represent latent variables which are represented in the structural equation analysis by multiple indicators. The squares are directly represented by values of scales designed to measure each construct.

## Methods

### Participants and procedure

A cross-sectional web-based study was conducted in mainland China from 19 May 2020 to 1 June 2020 during the COVID-19 outbreak. According to Jackson (2003), the number of participants for each parameter to be estimated should have to be 20 or greater. The number of parameters is 50 in this study, so at least (50 times 20=) 1,000 participants should have been included. Participants were recruited by the online WenJuanXing platform powered by WWW.wjx.cn *via* a snowball sampling method. Online informed consent was obtained from all participants and the responses were anonymous. The telephone number and email of the first author were attached at the beginning of the questionnaire in case participants had questionnaire-related questions. Participants were encouraged to forward a link or quick response code to other people they knew. The web survey required all participants to complete all items before submission and every participant was allowed to fill out only the questionnaire once due to preset equipment constraints, so that there were no missing data and repetitive filling out. The study procedure was approved by the Ethics Committee of the author's university (Approval No. E2020102).

The inclusion criteria for participation in this study were (1) being Chinese; (2) residing in mainland China (Considering that Hong Kong, Taiwan, and Macao belong to the special administrative regions of China, they are quite different from the mainland in terms of culture, policy, and economy). There were 1280 participants across China who participated in the survey. One hundred and thirteen participants were excluded, either because the time taken to answer the questionnaire was too short (< 120 s) or because the responses were logically contradictory. The research team conducted a pilot test in six people to make a realistic estimate of the time taken to complete the questionnaire. The result of the pilot study showed that the completion time was no less than 120 s. Data from 1,167 participants were included in this study with an effective response rate of 91.17%. The number of participants per parameter is approximately 23.

The descriptive statistics are shown in Table 1. The average age of the participants was 26.35 (SD = 10.05), with a range of 12–64 years. There were more female (76%) participants than male participants (24%). Most of the people (90%) did not have religion. Fifty-nine percent of participants lived in the urban area of China, while the remaining participants (41%) lived in a rural area. Many participants (69%) had a regular income. More than half of the participants (57%) had university or equivalent level of education. There were 44% of participants who had COVID-19 related sleep disturbance and approximately 56% of the participants who had COVID-19 related anxiety.

**Table 1:** Descriptive statistics for demographic characteristics, death anxiety, presence of meaning, search meaning, self-esteem, social support, COVID-19 related sleep disturbance, and COVID-19 related anxiety (*N* = 1167).

**Variables**	**#/Mean**	**%/(SD)**
Age		
≤ 24 year	700	59.98
25–34 year	282	24.16
35 year ≤	185	15.86
Gender		
Male	279	23.91
Female	888	76.09
Religion		
Yes	111	9.52
No	1,056	90.48
Current residence		
Urban area	686	58.78
Rural area	481	41.22
Income stability		
Regular	804	68.89
Variable	363	31.11
Educational background		
Middle school and below or equivalent experience	48	4.11
High school or equivalent experience	231	19.79
University or equivalent experience	665	56.98
Master and above	223	19.12
COVID-19 related sleep disturbance		
Yes	516	44.22
No	651	55.78
COVID-19 related anxiety		
Yes	648	55.53
No	519	44.47
Death anxiety	*M* = 7.75	(3.21)
Social support	*M* = 59.13	(11.81)
Presence of meaning	*M* = 23.11	(5.63)
Self-esteem	*M* = 28.88	(4.46)

### Instruments

#### Demographic characteristics

Age was calculated by subtracting the birth year from the survey year 2020. Gender (female = 1, male = 2), religion (yes = 1, no = 2), current residence (urban area vs. rural area), and income stability (regular vs. variable) were defined as binary variables. Educational background was categorized as middle school and below or equivalent experience, high school or equivalent experience, university or equivalent experience, and master and above, “Do you have COVID-19 related sleep disturbance?” (yes = 1, no = 2), “Do you have COVID-19 related sleep disturbance?” (yes = 1, no = 2) (Table 1). Age, gender, COVID-19 related sleep disturbance, and COVID-19 related anxiety were considered as control variables in our structural equation model (SEM) based on previous research showing associations with our core variables (Vittinghoff et al., 2011; Robah, 2017; Fernández et al., 2020).

#### Death anxiety scale (DAS)

The Chinese version (Zhang et al., 2019) of Templer's (1970) was used to assess death anxiety. It is a 15-item unidimensional self-reported instrument with the response options being “yes” or “no” (yes = 1; No = 0) (Templer, 1970). Six items are reverse scored. A total DAS score is the sum of the score of response to 15 items after reverse scoring and ranges from 0 to 15. A score ≤ 6 indicates mild death anxiety, a cut-off score between 7 and 9 indicates moderate death anxiety, and a score ≥10 indicates severe death anxiety (Mohammadi et al., 2022). In this study, the DAS showed acceptable reliability (Cronbach's alpha was 0.75).

#### The multidimensional scale of perceived social support (MSPSS)

The Chinese version (Zhou et al., 2015) of the MSPSS is a 12-item scale consisting of three dimensions, which are used to evaluate perceived support from three sources: family, friends, and significant others, with each item rated on a 7-point Likert scale (1 = strongly disagree to 7 = strongly agree) (Zimet et al., 1988). A total MSPSS score is the sum of responses to 12 items, which ranges from 12 to 84. Higher scores indicate higher levels of perceived support. Reliability of the MSPSS was good in the current sample with Cronbach's alpha being 0.93.

#### Presence of meaning scale (MLQ-P)

The Chinese version (Zhang et al., 2019) of presence of meaning scale is derived from the meaning in life questionnaire (MLQ) (Steger et al., 2006). Research has showed that presence of a meaning scale can be used as a unidimensional scale, which is a 5-item scale (Li et al., 2021b). Each item is rated using a 7-point Likert scale (1 = strongly disagree to 7 = strongly agree). The total MLQ-P score is the sum of responses to five items and ranges from 5 to 35. People with higher scores possess higher levels of presence of meaning (Steger et al., 2006). In this study, presence of meaning scale shows good internal consistency with a Cronbach's alpha of 0.85.

#### Rosenberg self-esteem scale (RSE)

The Chinese version (Tang et al., 2018) of the RSE is a 10-item unidimensional self-assessment scale that measures individual self-esteem. Each item was rated on a 4-point Likert scale (1 = strongly disagree to 4 = strongly agree) (Rosenberg, 1979). Four items are reverse scored. A total score is calculated by summing all the scores of responses after reverse scoring the negative worded relevant items and ranges from 10 to 40. In general, a score ≤ 25 indicates low self-esteem, a score between 26 and 32 indicates medium self-esteem, and a score ≥33 indicates high self-esteem (Rosenberg, 1979). In this study, the RSE has demonstrated acceptable reliability with a Cronbach's alpha was 0.86.

### Statistical analysis

Although there are cut-off thresholds for clinical relevance of the DAS and the RSE, our analysis focuses on the quantitative values of the scales in our implementation of the SEM. Descriptive statistics conducted by IMB SPSS Statistics version 21 (IBM Corp, 2012) are shown in Table 1. The total scores of death anxiety, social support, presence of meaning, and self-esteem were approximately normally distributed according to the QQ (quantile-quantile) plot. The characteristics of participants were described using frequencies with percentages. The Pearson correlations were used to investigate the correlations among social support, self-esteem, presence of meaning, and death anxiety. *P*-values < 0.05 determined statistical significance.

Confirmatory factor analysis (CFA), SEM, and pairwise contrasts were conducted in Mplus version 8.3 (Muthén and Muthén, 2017). When CFA and SEM were conducted, maximum likelihood estimation was applied. The method of 5,000 bootstraps was used to explore the significance of the mediation effect, which produces 95% bias-corrected confidence intervals (95% CIs). A “good fit” for the CFA and SEM suggested that the value of the comparative fit index (CFI) and Tucker–Lewis index (TLI) should be no lower than 0.90, and the value of the root mean square error of approximation (RMSEA) and standardized root mean square residual (SRMR) should be no higher than 0.08 (Hu and Bentler, 1999). The MODEL INDIRECT command was applied to test the standardized indirect effect presented in the multiple mediation model in this study (Cox et al., 2021). The MODEL CONSTRAINT command was used to examine whether the three indirect effects differed from each other statistically in pairwise contrasts. The difference was statistically significant, if 95% CI did not include zero.

## Results

### Descriptive statistics for death anxiety, social support, presence of meaning, and self-esteem

The scores for death anxiety (M = 7.75, SD = 3.21), social support (M = 59.13, SD = 11.81), presence of meaning (M = 23.11, SD = 5.63), and self-esteem (M = 28.88, SD = 4.46) are presented in Table 1. We found that average levels of death anxiety and self-esteem in our study were moderate (Rosenberg, 1979; Mohammadi et al., 2022).

### Correlation analysis

Pearson's correlation matrix for social support, presence of meaning, self-esteem, and death anxiety is provided in Table 2. Pearson's correlation analysis revealed the presence of meaning (*r* = −0.239, *p* < 0.010), self-esteem (*r* = −0.256, *p* < 0.010), and social support (*r* = −0.169, *p* < 0.010) negatively correlated with death anxiety. Presence of meaning was positively correlated with self-esteem (*r* = 0.591, *p* < 0.010) and social support (*r* = 0.429, *p* < 0.010). Self-esteem was positively correlated with social support (*r* = 0.482, *p* < 0.010).

**Table 2:** Pearson's correlation matrix for death anxiety, social support, presence of meaning, and self-esteem (*N* = 1,167).

	**Death**	**Presence of**	**Self-esteem**	**Social**
	**anxiety**	**meaning**		**support**
Death anxiety	1			
Presence of meaning	−0.239^**^			
Self-esteem	−0.256^**^	0.591^**^		
Social support	−0.169^**^	0.429^**^	0.482^**^	1

** *P* < 0.010 (two-tailed).

### SEM and interpretation

**Figure 2:**
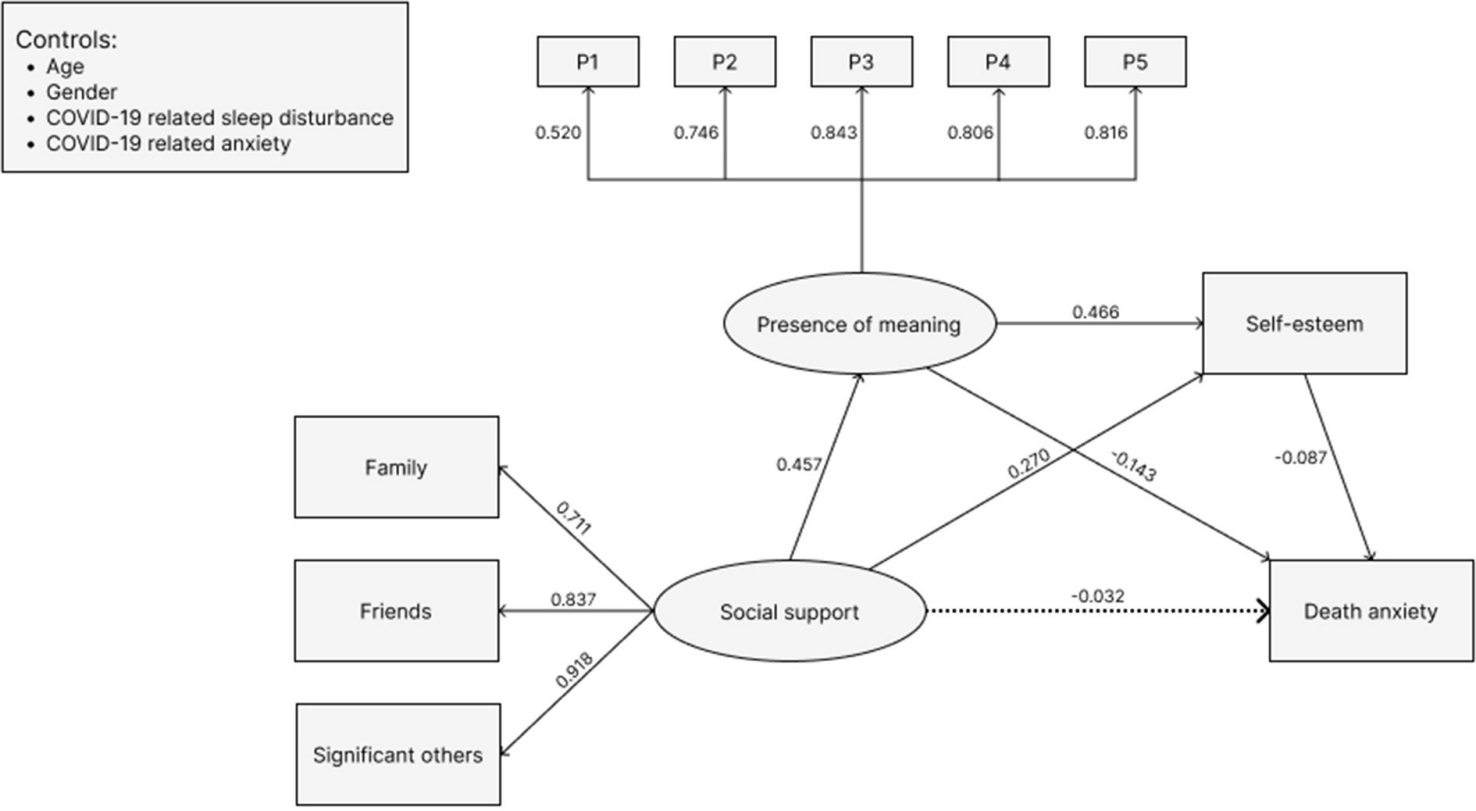
Final model for the whole sample (*N* = 1167), with standardized bate weights. Fit index: *x*^2^ = 243.384, df = 58, *p* < 0.001; CFI = 0.968, TLI = 0.954, RMSEA = 0.052, SRMR = 0.044. Circles indicate latent variables, while rectangles represent observed variables. Solid lines indicate paths are statistically significant while dashed line indicate paths are noit statistically significant. Abbreviations: Items P1–P5 measure presence of meaning.

The CFA of indices of social support is saturated: χ^2^ = 0, df = 0, *p* < 0.001, CFI = 1, TLI = 1, RMSEA = 0, SRMR = 0, and therefore fits the correlation matrix perfectly (Hu and Bentler, 1999). The fit of indices of presence of meaning is good: χ^2^ = 51.242, df = 5, *p* < 0.001, CFI = 983, TLI = 0.966, RMSEA = 0.089, SRMR = 0.024 (Hu and Bentler, 1999). The standard factor loadings in the measurement model of social support and presence of meaning are all significant and vary between 0.479 and 0.931, which are considered good (Wang, 2014), suggesting the items and observed variables were well explained by the latent variables (Wang, 2014). The final SEM is given in Figure 2, which shows a good fit for the multiple indicators and structural components of the full model: χ^2^ = 243.384, df = 58, *p* < 0.001; CFI = 0.968, TLI = 0.954, RMSEA = 0.052, SRMR = 0.044 (Hu and Bentler, 1999).

The unstandardized and standardized path coefficients for all pathways in the model of social support and death anxiety, mediated by presence of meaning, and self-esteem, are summarized in Table 3. The SEM was consistent with H1, H2, and H3 while controlling for covariates (age, gender, COVID-19 related sleep disturbance, and COVID-19 related anxiety). Results showed that a higher level of social support (β = −0.032, SE = 0.033), presence of meaning (β = −0.143, SE = 0.037), and self-esteem (β = −0.087, SE = 0.039) were associated with a lower level of death anxiety. A higher level of social support was associated with a greater level of presence of meaning (β = 0.457, SE = 0.033) and self-esteem (β = 0.270, SE = 0.032). A higher level of presence of meaning was associated with a greater level of self-esteem (β = 0.466, SE = 0.032). The relationships were all significant, except the direct effect from social support to death anxiety.

**Table 3:** The unstandardized and standardized path coefficients for all pathways in the model of social support and death anxiety, mediated by presence of meaning and self-esteem (*N* = 1,167).

	***b***	**B**	**S.E**	***t***	***P***
Social support → Presence of meaning (a1)	0.108	0.457	0.033	13.759	< 0.001
Social support → Self-esteem (a2)	0.344	0.270	0.032	8.340	< 0.001
Social support → Death anxiety (c)	−0.029	−0.032	0.033	−0.976	0.329
Presence of meaning → Self- esteem (d1)	2.500	0.466	0.032	14.381	< 0.001
Presence of meaning → Death anxiety (b1)	−0.553	−0.143	0.037	−3.853	< 0.001
Self- esteem → Death anxiety (b2)	−0.063	−0.087	0.039	−2.258	< 0.050

The standardized indirect effects in model of social support and death anxiety, mediated by presence of meaning, and self-esteem while controlling for covariates are presented in Table 4. The indirect effect of social support on death anxiety *via* presence of meaning was −0.065 (95% CI = −0.103, −0.032). The indirect effect of social support on death anxiety *via* self-esteem was −0.024 (95% CI = −0.047, −0.004). The indirect effect of social support on death anxiety *via* presence of meaning and self-esteem was −0.019 (95% CI = −0.037, −0.003). The results show that presence of meaning, self-esteem, and death anxiety can be explained by this model at 26.43%, 43.91%, and 23.74%, respectively.

**Table 4:** The standardized indirect effect in the model of social support and death anxiety, mediated by presence of meaning, and self-esteem.

**Path**	**Estimate**	**SE**	**95% CI**	
			**Lower**	**Upper**
Social support → Presence of meaning → Death anxiety (a1b1)	−0.065	0.018	−0.103	−0.032
Social support → Presence of meaning → Self-esteem → Death anxiety (a1d1b2)	−0.019	0.009	−0.037	−0.003
Social support → Self-esteem → Death anxiety (a2b2)	−0.024	0.011	−0.047	−0.004

The effect is statistically significant at *p* < 0.050 when the CI does not include zero. Abbreviations: CI, confidence interval.

The pairwise contrasts of the unstandardized indirect effects in model of social support and death anxiety, mediated by presence of meaning, and self-esteem are presented in Table 5. The results show that the mediation effect of presence of meaning was statistically different from the serial-multiple mediation effect of presence of meaning and self-esteem. Based on the comparison of indirect effects, the presence of meaning has a significantly stronger mediation than the serial-multiple mediation of presence of meaning and self-esteem. The effects of simple mediation between presence of meaning and self-esteem are not statistically significant (95% CI = −0.084, 0.005). The effects between simple mediation of self-esteem and the serial-multiple mediation of presence of meaning and self-esteem are also not statistically significant (95% CI = −0.018, 0.002).

**Table 5:** The pairwise contrasts of the unstandardized indirect effects in the model of social support and death anxiety, mediated by presence of meaning and self-esteem.

**Pairwise contrasts**	**Estimate**	**SE**	**95% CI**	
			**Lower**	**Lower**
Model 1 vs. Model 2	−0.038	0.023	−0.084	0.005
Model 1 vs. Model 3	−0.043	0.021	−0.005	−0.002
Model 2 vs. Model 3	−0.05	0.005	−0.018	0.002

The effect is statistically significant at *p* < 0.050 when the CI does not include zero. Abbreviations: CI, confidence interval; Model 1: Social support → Presence of meaning → Death anxiety; Model 2: Social support → Self-esteem → Death anxiety; Model 3: Social support → Presence of meaning → Self-esteem → Death anxiety; SE, standard error.

## Discussion

To the best of our knowledge, this study is one of the few empirical investigations to examine the relationships among social support, presence of meaning, self-esteem, and death anxiety based on the TMT. This study has contributed to the existing evidence that presence of meaning and self-esteem could separately and serially mediate the relationship between social support and death anxiety. Findings are consistent with H1, H2, and H3. Among indirect effects, presence of meaning has a stronger mediation than that of serial-multiple mediation of presence of meaning and self-esteem. Findings add to the knowledge of the relationships among social support, presence of meaning, self-esteem, and death anxiety based on TMT, and have practical implications for developing multicomponent interventions to manage death anxiety targeting increasing social support, presence of meaning, and self-esteem or increasing presence of meaning and self-esteem when social support is diminished.

The death anxiety in participants in this study during the pandemic period is higher than that in the elderly people during the non-pandemic period (Sinoff, 2017). One of the reasons that account for the high death anxiety level in this study could be the participants' age. The average age of the participants is approximately 26 years old at which death anxiety peaked (Chopik, 2017). Young adults in China in their 20s generally start taking over the family responsibility of raising their children and taking care of their aging parents under the influence of Chinese culture (Lee, 2015). They may have anxiety about whether their “deaths” burden the whole family as they think “if anything happened to me, what would happen to my family?” (Lee, 2015). The other reason for the high death anxiety level in this study is the COVID-19 pandemic (Pradhan et al., 2020). The death anxiety level of the general population examined in the COVID-19 pandemic was found to be much higher than that examined in the non-pandemic period (An et al., 2018). Observers have noted that the number of deaths and strong infectiousness of COVID-19 have very likely increased death anxiety (Rosenblatt et al., 1989; Pradhan et al., 2020).

This study also found that participants' perceived social support level was far less than the cancer patient, cancer patient's caregivers, and health providers (Liu and Aungsuroch, 2019; Uslu et al., 2019). More attention has been paid to cancer patients, cancer patient's caregivers, and health providers compared to the general population, and relatively comprehensive support systems (e.g., psychological consultation, palliative care) have been developed for these populations (West et al., 2016). The level of presence of meaning and self-esteem are similar to Lin's results tested in the non-pandemic situation (Lin, 2019). One reason that pre-pandemic and pandemic levels are similar could be that presence of meaning and self-esteem have high stability over time (Li et al., 2021b). In addition, TMT suggests that increased death anxiety would urge individuals to maintain their self-esteem and presence of meaning to buffer the anxiety (Greenberg et al., 2014). However, in the COVID-19 pandemic, people require more social support, presence of meaning, and self-esteem to protect against increasing death anxiety level (Zhang et al., 2019; He and Li, 2022). A more complete support system targeting increasing social support, presence of meaning, and self-esteem should be established for situations such as epidemics that by their nature reduce prospects for supportive social interactions.

Direct association between social support and death anxiety was reported before (Uslu et al., 2019; Bibi and Khalid, 2020). According to TMT and prior studies, supports from social network members play an important role in reducing death anxiety (Ebrahimi et al., 2018). In our study, a higher social support was associated with lower death anxiety in bivariate analyses. However, when adding and controlling for presence of meaning as predictor of self-esteem, and presence of meaning as well as self-esteem as predictors of death anxiety, social support was no longer related to death anxiety. This indicates presence of meaning and self-esteem fully mediate the relationship, respectively, and sequentially (Krause, 2007; Xia and Yang, 2019; Poudel et al., 2020). These mediators may capture the most important mechanisms at work in the relation between social support and death anxiety.

This study demonstrates that presence of meaning and self-esteem could fully mediate the relationship between social support and death anxiety which supports H2. To be specific, feeling being supported by social networking provides a deeper sense of meaning and the possibility for individuals to maintain their significance through a symbolic form of immortality, thus higher social support is associated with less death anxiety via increasing presence of meaning (Krause, 2007; Holt, 2018). Additionally, as TMT suggests, social support could provide a sense of personal value and security which contribute to forming positive thoughts about oneself and lead to a high level of self-esteem (Greenberg et al., 2014), thus higher social support is associated with less death anxiety *via* increasing self-esteem (Poudel et al., 2020). Previous studies have consistently reported that social support increases the level of presence of meaning and self-esteem, and that presence of meaning and self-esteem reduce death anxiety (Ebrahimi et al., 2018; Zhang et al., 2019; He and Li, 2022). However, previous studies have seldom considered the four variables in one multiple mediation model. This study provides empirical evidence for the relationships among social support, presence of meaning, self-esteem, and death anxiety based on TMT.

This study uniquely revealed the sequential mediation of presence of meaning and self-esteem between the relationship of social support and death anxiety. The sequential mediation of self-esteem and meaning in life in Lin's multiple mediation model differs from but does not contradict our results. In Lin's study (2019), self-esteem and meaning in life mediated the relationship of gratitude and suicidal ideation sequentially. People with a greater level of self-esteem tend to foster meaning in life through making people feel valued and important. In our research, having a meaningful life increases the feeling of self-worth, the positive emotion generated when individuals feel that their talents and personality are valued (Martela and Steger, 2016), and these emotions could boost self-esteem (Martela and Steger, 2016; Barnett et al., 2019). Although previous research implies the potential bidirectional association between presence of meaning and self-esteem (Barnett et al., 2019; Lin, 2019; Zhang et al., 2019), the relationship of presence of meaning and self-esteem needs to be discussed in the context of specific research purposes (Greenberg et al., 2014).

Another interesting finding we come across in this study is that presence of meaning had a stronger mediation than the serial-multiple mediation of presence of meaning and self-esteem. This demonstrates presence of meaning is important for death anxiety when increasing social support, more than the multiple influences of presence of meaning and self-esteem. Lin has pointed a similar result that meaning in life played a more important role than self-esteem in the relationship of gratitude and suicidal ideation, but the difference between the single mediation of meaning in life and the sequential multiple mediation of self-esteem and meaning in life was not examined (Lin, 2019). Presence of meaning is a broader concept compared with self-esteem. It represents a cluster of related factors, for instance, having significance, broader purpose, and certainty and comprehensibility in life, all of which transmit the effect of social support to death anxiety (Krause, 2007; Martela and Steger, 2016), thus presence of meaning buffers death anxiety directly and effectively (Krause, 2007; Greenberg et al., 2014; Holt, 2018).

## Implications

This research has profound theoretical and practical implications. Findings examine the relationships among social support, presence of meaning, self-esteem, and death anxiety based on the TMT and provide an understanding of death anxiety and placing more importance on developing multicomponent interventions targeting increasing social support, presence of meaning and self-esteem, or increasing presence of meaning and self-esteem when social support is diminished in the pandemic. Moreover, the result of this study also provides an empirical evidence for the TMT in the context of COVID-19 pandemic, and also suggestions for future research.

First, strategies to improve social support are critical to buffer death anxiety when mortality is made salient, especially in the time of COVID-19. Joint efforts by government, social workers, health professionals as well as affected individuals are required to address these issues (Krause, 2007; Plusnin, 2018). Given the recommendation to follow minimum face-to-face intervention, a web-based public health emergency management system should be developed (Liang and Xue, 2004; Cao et al., 2020). Resources for daily necessities should be guaranteed and up-to-date information such as providing abundant information regarding disease itself, online courses for self-protection skills and health education, and online platforms for medical-care-seeking and entertainments systematically should be easily accessed. In addition, though physical distancing was forcibly required, mutual communications and emotional supports between individuals *via* telecommunication techniques to increase interactions are effective (Krause, 2007).

Second, meaning-oriented programs may be beneficial to individuals during the period of the COVID-19 pandemic. For instance, in a customized intervention called Life Crafting, people rediscover meaning by making sense of what has happened to them and trying to focus on their ideal future *via* online writing exercises through four steps (Dejong et al., 2020). Further meaning-oriented programs catering to Chinese culture should be developed (Li et al., 2021a).

Third, interventions increasing self-esteem should be taken into consideration by communities and individuals. Cognitive-behavioral therapy (CBT)-based programs have been proved to enhance self-esteem effectively (Terp et al., 2019; Lee et al., 2020). Further studies regarding CBT-based interventions in accordance with China's national condition should be conducted.

## Limitation

First, participants were recruited online via a non-probabilistic snowball sampling method, and they were restricted to people who used the internet. Where possible, we conducted analysis of the main relationships in subsamples of the main sample. Because the sample was largely female, the results reflect the views of women more than of men. And in the small sample of men, we found that social support (with controls on the other factors) was a significant and direct protective factor for death anxiety and self-esteem, in contrast to the results for women, was not significant. These intriguing differences await study in a larger and more representative sample. Second, the TMT also suggests that increased death anxiety would urge individuals to maintain their self-esteem and presence of meaning to buffer the anxiety. In this regard, there are potential bidirectional associations between death anxiety and self-esteem and between death anxiety and meaning in life. It would be useful and promising to conduct longitudinal studies to analyze the dynamic interrelationships of social support, presence of meaning, self-esteem, and death anxiety in the future.

## Conclusion

Overall, this study provides insights into interrelationships between social support, presence of meaning, self-esteem, and death anxiety based on the TMT: presence of meaning and self-esteem fully mediated the relationship between social support and death anxiety, respectively, and sequentially. Moreover, presence of meaning had a stronger mediation than the serial-multiple mediation of presence of meaning and self-esteem. This study provides a perspective on the mechanism of how social support, presence of meaning, and self-esteem work to buffer death anxiety. Findings provide an understanding of death anxiety and emphasize the importance of developing multicomponent interventions targeting increasing social support, presence of meaning, and self-esteem and increasing presence of meaning and self-esteem when social support is diminished in the pandemic.

## Data availability statement

The raw data supporting the conclusions of this article will be made available by the authors, without undue reservation.

## Ethics statement

The studies involving human participants were reviewed and approved by Ethics Committee of Xiang Ya School of Nursing, Central South University. Written informed consent from the participants' legal guardian/next of kin was not required to participate in this study in accordance with the national legislation and the institutional requirements.

## Author contributions

YH, NR, ST, and MS contributed to the conception and design of the study. YH and MS organized the database and performed the statistical analysis. YH wrote the first draft of the manuscript. YH, ZG, FY, JW, ST, and MS contributed to the manuscript revision. All authors have read and approved the submitted version.

## Funding

This research was supported by the Research Project of Teaching Reform in Colleges and Universities of Hunan Province under Grant Number HNJG-2022-0449.

## Conflict of interest

The authors declare that the research was conducted in the absence of any commercial or financial relationships that could be construed as a potential conflict of interest.

## Publisher's note

All claims expressed in this article are solely those of the authors and do not necessarily represent those of their affiliated organizations, or those of the publisher, the editors and the reviewers. Any product that may be evaluated in this article, or claim that may be made by its manufacturer, is not guaranteed or endorsed by the publisher.
